# N6-Methyladenosine Methyltransferase Component KIAA1429 Is a Potential Target of Cancer Therapy

**DOI:** 10.3390/biom14101319

**Published:** 2024-10-17

**Authors:** Junjun Huang, Jihua Guo, Rong Jia

**Affiliations:** 1 State Key Laboratory of Oral & Maxillofacial Reconstruction and Regeneration, Key Laboratory of Oral Biomedicine Ministry of Education, Hubei Key Laboratory of Stomatology, School & Hospital of Stomatology, Wuhan University, Wuhan 430072, China; junjunhuang@whu.edu.cn (J.H.); jihuaguo@whu.edu.cn (J.G.); 2 Department of Endodontics, School & Hospital of Stomatology, Wuhan University, Wuhan 430072, China

**Keywords:** KIAA1429, m^6^A modification, tumorigenesis, cancer therapy

## Abstract

N6-methyladenosine (m^6^A), the most abundant RNA modification in eukaryotes, has a crucial impact on tumorigenesis. KIAA1429 is the key component of the m^6^A methyltransferase complex, in which KIAA1429 functions as a scaffold to bridge the catalytic core proteins. KIAA1429 is often overexpressed in malignances, associated with patient prognosis, and required for tumorigenesis. KIAA1429 regulates the expression of a number of tumor-associated genes in an m^6^A -dependent manner, and thus, contributes to cell proliferation, migration, drug resistance, tumor formation and metastasis. This review focuses on recent progress in the understanding of roles and mechanisms of KIAA1429 in cancers, and offers ideas for potential anti-cancer therapeutic methods by targeting KIAA1429.

## 1. Introduction

N^6^-methyladenosine (m^6^A) at the N^6^ position of adenosine is the most abundant modification in eukaryotic messenger RNAs. The deposition of m^6^A is a dynamic process involving a series of factors, including “writers” and “erasers” to adjust the levels of m^6^A modification in RNAs continuously. Many RNA-binding proteins are involved in the recognition of m^6^A modification in RNAs as “readers”, and affect an RNA’s stability, alternative splicing, translation, and so on. Accumulated evidence has revealed the important roles of m^6^A modification in tumorigenesis [1].

KIAA1429, as a writer (also called VIRMA, vir-Like m^6^A methyltransferase associated, virilizer), is a key component of the methyltransferase complex. It is the biggest protein in the complex, bridges the catalytic core parts of the methyltransferase complex as a scaffold [2], and recruits METTL3 (methyltransferase-like 3), METTL14 (methyltransferase-like 14), and WTAP (Wilms’ tumor 1-associating protein) to specific sites of m^6^A modification [3]. Knockdown of KIAA1429 significantly down-regulated the m^6^A modification of cellular RNA [4]. In addition to m^6^A modification, KIAA1429 can also regulate many other cellular processes, such as alternative polyadenylation, alternative splicing, ferroptosis, and so on. KIAA1429 is associated with polyadenylation cleavage factors CPSF5 (cleavage and polyadenylation specificity factor 5) and CPSF6 (cleavage and polyadenylation specificity factor 6) in the presence of RNA, and promotes the usage of a proximal polyadenylation site [3].

In this review, we focus on recent progress in the understanding of the roles and mechanisms of KIAA1429 in cancers, as well as the potential anti-cancer therapeutic methods targeting KIAA1429. In addition, the characteristics of KIAA1429′s gene, its protein, its alternative splicing, and the implications of these characteristics in cancers are discussed comprehensively. To substantially understand the roles of KIAA1429 in cancers, this review mainly focuses on the experimental studies in the field.

## 2. Gene and Protein Structure of KIAA1429

**Figure 1:**
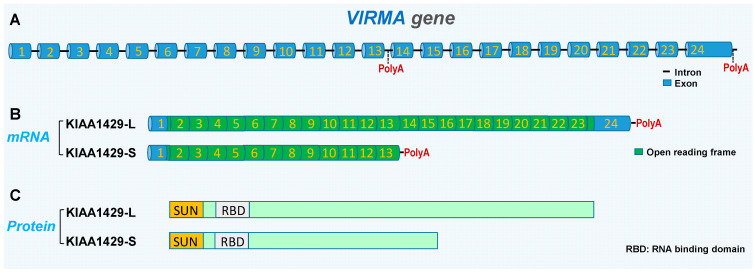
Gene structure and isoforms of the *VIRMA* gene encoding the KIAA1429 protein. (**A**) The gene structure of the *VIRMA* gene. (**B**) The mRNA structures of the KIAA1429 long isoform (KIAA1429-L) and the KIAA1429 short isoform (KIAA1429-S). (**C**) The structures of proteins encoded by KIAA1429 isoforms.

The *VIRMA* gene encodes the KIAA1429 protein in human. According to the data in Genbank, it has at least two isoforms generated by alternative polyadenylation site selection. A long isoform (KIAA1429-L) contains 24 exons and encodes an 1812-amino acid (aa) protein. A short isoform (KIAA1429-S) contains 13 exons by using a proximal polyadenylation site and encodes a shorter 1147-amino acid protein. Function domains of KIAA1429 are not clear. Both the long and short KIAA1429 protein have a 130-aa SUN domain at the beginning, which is an RNA-binding domain (aa 155–279) [5] (Figure 1). So far, the function of the KIAA1429 short isoform and the regulatory mechanism of the alternative selection of polyadenylation sites remains largely unknown. It contains the N-terminal of the full-length KIAA1429 protein, and the N-terminal is responsible for recruiting catalytic core members METTL3, METTL14, and WTAP of methyltransferase [3], suggesting this short isoform may have roles in m^6^A modification. However, more research is required for exploring the function of the KIAA1429-S protein, which lacks almost all of the C-terminal region.

## 3. Expression and Clinical Significance of KIAA1429 in Cancers

Many studies have reported the overexpression of KIAA1429 in the transcriptional levels in different types of cancer by using bioinformatics analyses. Because the KIAA1429 protein plays substantial roles, it is essential to understand the features of KIAA1429 protein expression in cancers. Recently, the expression levels of the KIAA1429 protein have been investigated in the independent cohorts of different cancers. In liver cancer, the KIAA1429 protein is overexpressed in tumor tissues compared with adjacent normal tissues [6,7]. In lung adenocarcinoma, a high KIAA1429 protein expression level is significantly associated with disease progression and poor prognosis [8]. In ovarian cancer, tumor tissues expressed significantly higher KIAA1429 protein amounts [9]. In diffuse large B-cell lymphoma (DLBCL), the KIAA1429 protein expression level is significantly higher than in the reactive hyperplasias of lymph nodes, and is positively associated with poor overall survival in DLBCL patients [10]. In colorectal adenocarcinoma, the KIAA1429 protein was overexpressed in tumor tissues compared with normal tissues [11,12], and was also associated with the poor overall survival of patients [12]. The KIAA1429 protein is also significantly overexpressed in non-small cell lung cancer [13]. Multiple myeloma is a malignancy originated from plasma cells. Multiple myeloma cells expressed a significantly higher level of the KIAA1429 protein than normal plasma cells, and patients with high levels of KIAA1429 showed poor overall survival [14]. KIAA1429 is also overexpressed in other cancers, such as oral squamous cell carcinoma [15] and gastric cancer [16]. Interestingly, the KIAA1429 protein was also overexpressed in some benign tumors. For example, infant hemangioma expressed significantly higher levels of the KIAA1429 protein than normal skin tissue [17].

The subcellular distribution of the KIAA1429 protein is associated with tumorigenesis. In breast cancer, a study showed that KIAA1429 was mainly localized in the nucleus of normal breast tissue, whereas most of tumor samples showed a predominantly cytosolic localization of KIAA1429, which suggested that KIAA1429 can affect the stability of its targets in cytoplasm [18].

Because KIAA1429 promotes the m^6^A modification of RNAs in many target transcripts, KIAA1429 overexpression will increase the overall m^6^A modification of cellular RNAs. Its overexpression and association with patients’ poor prognoses in cancers suggests that the KIAA1429 protein and the increased m^6^A modification play positive roles during tumorigenesis. However, whether KIAA1429 overexpression and high m^6^A modification can initiate tumorigenesis is unclear.

## 4. Roles of KIAA1429 in Cancers

**Figure 2:**
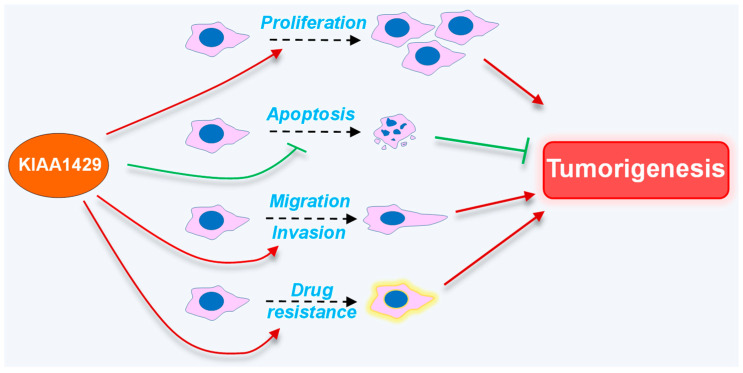
Roles of KIAA1429 in tumorigenesis.

To understand the function of KIAA1429 in cancers, a number of studies have investigated the roles of KIAA1429 in the cellular processes related to tumorigenesis, including cell proliferation, cellular apoptosis, migration and invasion, as well as drug resistance and in vivo tumor formation and metastasis (Figure 2).

### 4.1. Cell Proliferation

A number of studies have shown that the silence of KIAA1429 expression inhibited cell proliferation in many types of cancer cells, including liver cancer [7,19], gastric cancer [16,20], osteosarcoma [21], Ewing sarcoma [22], lung cancer [13,23,24], colorectal adenocarcinoma [11], ovarian cancer [9], and multiple myeloma [14]. Importantly, a study showed that KIAA1429 knockdown reduced the growth of breast cancer cells, but did not affect the growth of normal breast epithelial cells and 293T cells [18]. Moreover, a study knocked out KIAA1429 with the CRISPR/Cas9 method in diffuse large B-cell lymphoma and found that cell proliferation was significantly suppressed in KIAA1429 knockout cells [10]. The reduced cell proliferation is correlated with reduced Ki67 expression in both RNAs and protein levels upon KIAA1429 knockdown in Ewing sarcoma [22]. Consistently, some studies have demonstrated that the overexpression of KIAA1429 promoted cell proliferation in lung adenocarcinoma [23], diffuse large B-cell lymphoma [10], and chronic myeloid leukemia [25]. Therefore, up-regulated KIAA1429 expression plays an important function in supporting unlimited cancer cell proliferation.

### 4.2. Cellular Apoptosis

Cancer cells can escape apoptosis. Overexpression of KIAA1429 reduced apoptosis at least in diffuse large B-cell lymphoma [10] and chronic myeloid leukemia [25]. In contrast, KIAA1429 knockdown induced significant cellular apoptosis in many cancer cells, including liver cancer [19,26], osteosarcoma [21], Ewing sarcoma [22], lung adenocarcinoma [23], diffuse large B-cell lymphoma [10], ovarian cancer [9], and chronic myeloid leukemia [25]. The inhibition of KIAA1429 may be a promising method to induce cancer cell apoptosis.

### 4.3. Migration and Invasion

The ability to migrate and invade is an important characteristic of cancer cells. Studies have shown that KIAA1429 could promote the migration and invasion of cancer cells in breast cancer [5], lung adenocarcinoma [23,24], colorectal adenocarcinoma [11], gastric cancer [16], ovarian cancer [9], and chronic myeloid leukemia [25], indicating that KIAA1429 may be involved in the progress of multiple cancers.

### 4.4. Drug Resistance

Cisplatin acts as an anti-proliferation drug by inducing DNA damage in cancer cells. Cisplatin treatment increased KIAA1429 protein expression. The knockdown of KIAA1429 sensitized gastric cancer cells to cisplatin treatment by increasing oncogene FOXM1 (Forkhead box M1) expression [27]. Interestingly, another study revealed that KIAA1429 induced a resistance to oxaliplatin, another anti-proliferation drug, by inhibiting DNA synthesis and increasing FOXM1 expression [28]. KIAA1429 can promote the resistance to gefitinib, an EGFR (epidermal growth factor receptor) inhibitor, by increasing MAP3K2 (mitogen-activated protein kinase kinase kinase 2) expression in lung adenocarcinoma [29], or by suppressing autophagy mediated by WTAP in non-small cell lung cancer [30]. Imatinib is a tyrosine kinase inhibitor for the treatment of chronic myeloid leukemia. The overexpression of KIAA1429 significantly increased the resistance of chronic myeloid leukemia cells to imatinib [25]. KIAA1429 is able to promote the resistances of cancer cells to multiple types of anti-cancer drugs. It is valuable to illustrate the mechanisms of how the KIAA1429 protein increases resistances to different drugs and paves the way for treating the drug resistance of cancers.

### 4.5. Tumor Formation and Metastasis

In vivo tumor formation and metastasis experiments provide substantial evidence of KIAA1429′s roles in cancers. Many studies have performed in vivo tumor formation or metastasis experiments in different models.

Stable knockdown of KIAA1429 significantly reduced tumor formation in gastric cancer cells [16,20], osteosarcoma [21], lung cancer [13,29,31], live cancer [26], and multiple myeloma [14] in nude mice. KIAA1429 is required for cancer cell metastasis in vivo in liver cancer [6,19]. In breast cancer, knockdown of KIAA1429 reduced lung metastasis and prolonged the survival time in nude mice [5]. Stable knockdown of KIAA1429 showed a very strong inhibition of tumor growth and the lung metastasis of ovarian cancer cells in nude mice [9].

KIAA1429 knockout study provided further important evidence of its roles in tumorigenesis. By combining both in vitro and in vivo CRISPR-Cas9 knockout screening, KIAA1429 was identified as the key driver protein of Ewing sarcoma. By using an inducible anti-KIAA1429 shRNA expression system, this study further showed that induced knockdown of KIAA1429 significantly blocked tumor growth in nude mice [22].

In addition to nude mice, the down-regulation of KIAA1429 expression also attenuated tumorigenesis in other types of immunodeficient mice. For example, KIAA1429 knockout significantly reduced the tumor growth of diffuse large B-cell lymphoma cells in severe combined immunodeficiency beige mice [10]. KIAA1429 knockdown significantly reduced the tumor growth of colorectal cancer cells in severe combined immunodeficiency NOD/SCID mice [11]. KIAA1429 knockdown significantly reduced the tumor growth of breast cancer cells in immunodeficient NSG mice [18].

In summary, the knockdown or knockout of KIAA1429 attenuates tumorigenesis in multiple cancers. However, it remains unknown whether KIAA1429 overexpression can transform cells or enhance tumorigenesis. M^6^A is essentially a reversible modification in RNAs. Although the decrease in KIAA1429 and m^6^A modification can reduce tumor formation and the metastasis of cancer cells, the dynamic changes and roles of m^6^A modification and KIAA1429 expression during malignant cell transformation need further study.

## 5. Molecular Mechanisms of KIAA1429 in Cancers

**Figure 3:**
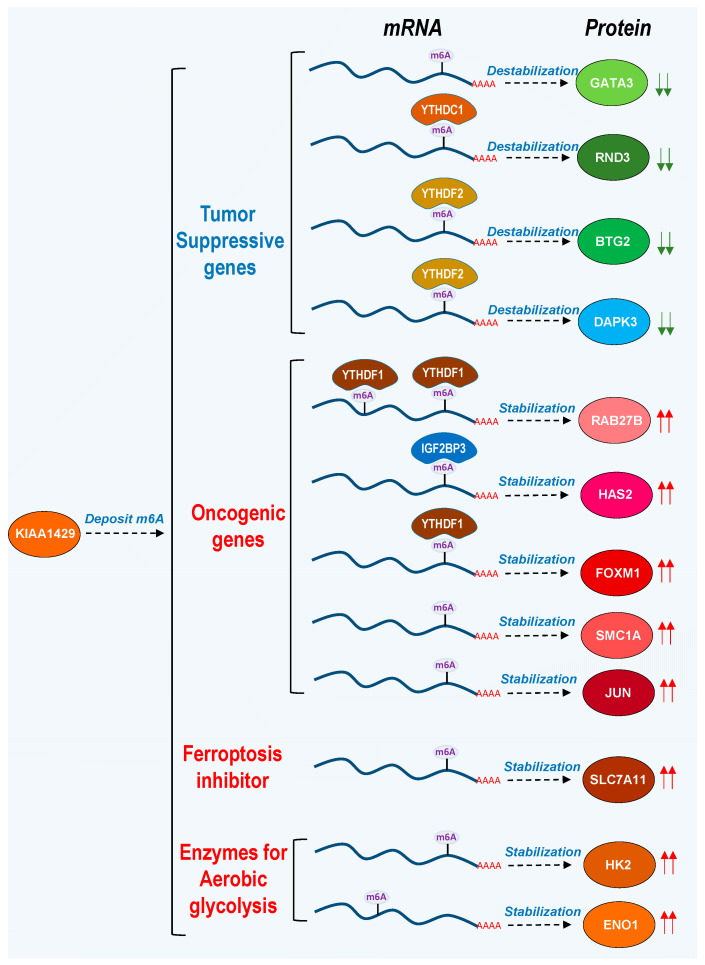
Molecular mechanisms of KIAA1429 in cancers. KIAA1429 destabilizes the mRNAs of some tumor-suppressive genes, including *GATA3*, *RND3*, *BTG2*, and *DAPK3*, but stabilizes the mRNAs of some oncogenic genes, such as *RAB27B*, *HAS2*, *FOXM1*, *SMC1A*, and *JUN*. In addition, KIAA1429 can stabilize the mRNAs of the *SLC7A11* gene, a ferroptosis inhibitor, and some enzymes for aerobic glycolysis, like *HK2* and *ENO1*. The down-arrow represents the downregulation of the protein level. The up-arrow represents the upregulation of the protein level.

KIAA1429 binds to its target RNAs and installs an m^6^A modification, which can increase or decrease the stability of target RNAs depending on the associated m^6^A reader proteins. Most of studies showed that KIAA1429 increases the stability of oncogenic genes and decreases the stability of tumor-suppressive genes, and then promotes tumorigenesis (Figure 3) (Table 1).

### 5.1. Down-Regulation of Tumor-Suppressive Genes

GATA3 (GATA Binding Protein 3) acts as a tumor suppressor by activating the transcription of E-cadherin and inhibiting the epithelial–mesenchymal transition (EMT) and metastasis [36]. KIAA1429 promotes m^6^A modification in the 3′-UTR of *GATA3* pre-mRNAs and prevents the association between the RNA-binding protein HuR and *GATA3* pre-mRNAs, and then leads to the instability of *GATA3* pre-mRNAs and the down-regulation of GATA3 protein expression. Notably, an antisense long noncoding RNA of GATA3, GATA3-AS, can interact with KIAA1429 and guide it to *GATA3* pre-mRNAs by base-pairing with the 5′ part of *GATA3* pre-mRNAs [19].

RND3 (Rho family GTPase 3) was considered as an anti-metastatic protein [37]. KIAA1429 increases the m^6^A methylation in 3′-UTR of the *RND3* mRNA and decreases its stability via m^6^A reader YTHDC1 (YTH N6-methyladenosine RNA binding protein C1), and then down-regulates the RND3 expression level in liver cancer [6].

BTG2 (B-cell translocation gene 2) can protect cells from oncogenic transformation by maintaining cellular homeostasis under stress [38]. KIAA1429 knockdown significantly up-regulated BTG2 expression, whereas KIAA1429 overexpression slightly down-regulated BTG2 levels in lung adenocarcinoma. BTG2 overexpression abolished the promotion of cell proliferation by KIAA1429. KIAA1429 promotes m^6^A modification in *BTG2* mRNA and leads to its instability mediated by YTHDF2 (YTH N6-methyladenosine RNA binding protein F2) [23].

RXFP1 (Relaxin family peptide receptor 1) is a receptor and signaling mediator of relaxin [39]. It inhibits the cell proliferation, migration, and invasion of lung cancer cells. KIAA1429 promoted lung cancer by down-regulating RXFP1 expression in an m^6^A-dependent manner in lung cancer [24].

P53 is a well-known tumor suppressor. It plays essential roles in monitoring DNA damage and genomic stability [40]. Loss of p53 in mice induced spontaneous tumor formation. Phosphorylation activates the p53 protein to induce apoptosis [41]. KIAA1429 knockdown promoted the phosphorylation of the p53 protein and activated downstream pathways, and then led to the inhibition of cell proliferation in non-small cell lung cancer [31].

DAPK3 (death-associated protein kinase 3) belongs to the death-associated protein kinase family. It is often down-regulated in cancers and can regulate programmed cell death [42]. KIAA1429 suppressed DAPK3 expression by installing m^6^A on its mRNA 3′-UTR and destabilizing its mRNA by YTHDF2 [13].

ARHGAP30 (Rho GTPase activating protein 30) activates p53 by facilitating the acetylation of p53 at lysine 382 [43]. KIAA1429 repressed ARHGAP30 expression by destabilizing its mRNA stability. The silence of ARHGAP30 partially rescued the suppressed proliferation and migration due to KIAA1429 knockdown [32].

KIAA1429 increases m^6^A modification in the transcripts of these tumor-suppressive genes, and leads to the instability of their transcripts via different m^6^A reads and the down-regulation of their mRNA and protein levels. Therefore, inhibiting KIAA1429 expression or function may rescue the expression of these genes and suppress tumorigenesis.

### 5.2. Up-Regulation of Oncogenic Genes

RAB27B (Ras-related protein Rab-27B) is a small GTPase of an Ras-related protein and regulates exocytosis of different types of vesicles, including exosome to inhibit senescence [44]. RAB27B expression is positively regulated by KIAA1429 via depositing m^6^A modifications in both coding and 3′-UTR regions of its mRNA. YTHDF1 (YTH N6-methyladenosine RNA binding protein F1) can recognize the m^6^A modification and increase its mRNA stability [25].

KIAA1429 binds to the 3′-UTR of *c-Jun* mRNA and stabilizes it, a process that is probably mediated by m^6^A modification [20]. In breast cancer, KIAA1429 promotes the epithelial–mesenchymal transition (EMT) by increasing SMC1A (structural maintenance of chromosomes 1A) expression in an m^6^A modification-dependent manner, and indirectly promoting SNAIL (Snail family transcriptional repressor 1) expression [5].

LncRNA POU6F2-AS1 (POU6F2 antisense RNA 1) is overexpressed in colorectal cancer and required for cell proliferation and invasion. KIAA1429 promotes POU6F2-AS1 expression in an m^6^A-dependent manner [34]. SIRT1 is another target of KIAA1429 in colorectal cancer. KIAA1429 deposits m^6^A to SIRT1 mRNA and increases its stability [11].

FOXM1 is a critical transcriptional factor that promotes cell proliferation by up-regulating DNA replication, cell cycle progression, and key factors of mitosis [45]. KIAA1429 binds to an m^6^A site of “AGGGACU” in the 3′-UTR of FOXM1 mRNA and increases its stability that is mediated by YTHDF1 [27]. Another study confirmed this regulation mechanism in multiple myeloma [14].

HAS2 (hyaluronan synthase 2) can promote angiogenesis and metastasis. The KIAA1429 protein interacts with the IGF2BP3 (insulin-like growth factor 2 mRNA-binding protein 3) protein, and then binds to and stabilizes m^6^A-modified HAS2 mRNA in breast cancer [18].

SOX8 (SRY-box transcription factor 8) can transactivate oncogene expression. KIAA1429 improves SOX8 expression by increasing its mRNA stability [35].

### 5.3. Ferroptosis

Ferroptosis is a type of programmed cell death characterized by the increased amount of ROS and lipid peroxidation, and damage in mitochondria. In hepatocellular carcinoma cells, KIAA1429 knockdown induced the increased intracellular ROS (reactive oxygen species) level and ferroptosis. The inhibition of ferroptosis rescued the cell proliferation suppressed by KIAA1429 knockdown. KIAA1429 promotes the expression of SLC7A11 (solute carrier family 7 member 11), a suppressor of ferroptosis, by binding to *SLC7A11* mRNA and increasing m^6^A modification. SLC7A11 overexpression rescued the tumor growth retardation caused by KIAA1429 knockdown [26]. In non-small cell lung cancer, KIAA1429 knockdown enhanced erastin-induced ferroptosis by increasing ROS levels [31]. Therefore, like many oncoproteins, KIAA1429 can suppress both apoptosis and ferroptosis to enhance the survival of cancer cells.

### 5.4. Aerobic Glycolysis

Aerobic glycolysis is a hallmark of many cancers. KIAA1429 can promote aerobic glycolysis including increasing glucose uptake and lactate production by up-regulating HK2 (hexokinase 2, a crucial enzyme in aerobic glycolysis) expression in an m^6^A-dependent manner in colorectal cancer [33]. Another study also demonstrated a similar phenotype in multiple myeloma [14]. HK1 is another important member of the hexokinase family. In liver cancer, KIAA1429 increases the m^6^A modification and stability of *HK1* mRNA, and leads to enhanced aerobic glycolysis [7]. In ovarian cancer, KIAA1429 also promotes aerobic glycolysis by depositing m^6^A in the “GGACU” motif of the open reading frame region of *ENO1* (enolase1, a glycolysis enzyme) mRNA and increasing its expression [9].

### 5.5. Telomere Length

Telomeres are essential for genome stability. Short telomeres suppress tumorigenesis. A genome-wide association study (GWAS) showed that KIAA1429 is genetically associated with telomere length. KIAA1429 knockdown in HTR8/Svneo, a transformed extravillous trophoblast cell line, induced significant telomere shortening [46]. This result suggested that KIAA1429 promotes tumorigenesis by maintaining telomere length in tumor cells. However, the related mechanism needs further exploration.

### 5.6. Targeted Pathways Identified by High-Throughput Methods

Many studies discussed in this review performed RNA-seq analyses to search potential targets in all kinds of cancers. In line with its oncogenic function, the knockdown of KIAA1429 often led to the significant enrichment of its targets in cell cycle, cell death, and apoptosis pathways [19]. As it is an RNA-binding protein, the knockdown of KIAA1429 also affected RNA-specific processes such as RNA modification, RNA transport, location, translation and metabolic process [19,23]. The potential effects of KIAA1429 on the alternative splicing of RNA were investigated in hepatocellular carcinoma cells [47]. Interestingly, intron retention is the major affected type of alternative splicing. KIAA1429 binds to many genes with affected events of alternative splicing, which are also highly enriched in cell cycle- and apoptosis-related pathways [47].

In particular cancers, KIAA1429 can regulate some specific pathways. In gastric cancer cells, KIAA1429 tends to regulate immune-associated pathways, and the TNF (tumor necrosis factor) signaling pathway is the pathway most affected by KIAA1429 [20]. In Ewing sarcoma, the knockdown of KIAA1429 induced significant changes in the cancer-associated inflammation pathway [22]. In non-small cell lung cancer, KIAA1429 knockdown is associated with galactose metabolism, mineral absorption, nicotine addiction, p53 signaling, and ferroptosis [31]. Another lung cancer study showed the stable silencing of the KIAA1429 affected the calcium, NF-kB, and IL-17 (interleukin-17) signaling pathway [13]. 

Some studies used both MeRIP-seq (methylated RNA immunoprecipitation sequencing) and RNA-seq (RNA sequencing) methods to explore KIAA1429′s target genes with significantly decreased m^6^A peaks and changed expression levels. A study performed these two methods discovered that the MAPK (mitogen-activated protein kinase) signaling pathway was the most significantly regulated pathway, and MAP3K2 (mitogen-activated protein kinase kinase kinase 2) was the direct downstream target of KIAA1429 [29]. KIAA1429 promotes cell proliferation and invasion by increasing MAP3K2 expression [29]. Another study of diffuse large B-cell lymphoma showed that m^6^A sites related to KIAA1429 are most enriched in coding regions and 3′UTR regions. The combination of MeRIP-seq and RNA-seq analyses, and gain-of-function and loss-of-function assays showed that carbohydrate sulfotransferase 11 (*CHST11*) is a true target of KIAA1429. KIAA1429 installed an m^6^A modification to *CHST11* mRNA, and then recruited YTHDF2 to decrease its stability and CHST11 protein expression [10]. In chronic myeloid leukemia, the combination of RNA-seq data and MeRIP-seq data in the m6a2target database showed that targets of KIAA1429 were mainly enriched in enzyme activity inhibition, focal adhesion, and exocytosis [25].

## 6. The Regulation of KIAA1429 Expression

**Figure 4:**
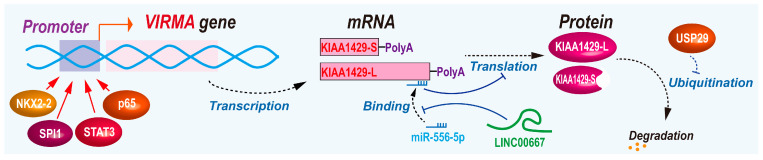
The regulation of KIAA1429 expression.

The regulatory mechanisms of KIAA1429 expression have been gradually revealed (Figure 4). A study showed that the knockdown of splicing factor TRA2A (transformer 2 alpha homolog) significantly reduced KIAA1429 protein expression in esophageal cancer [48]. In Ewing sarcoma, oncogenic transcription factor NKX2-2 (NK2 homeobox 2) transactivates KIAA1429 expression. Then, increased KIAA1429 can up-regulate STAT3 expression; in turn, STAT3 also can promote KIAA1429 expression, which forms positive feedback [22]. The NKX2-2/KIAA1429/STAT3 pathway is a new epigenetic/transcription regulatory pathway in cancer. More studies are required to confirm this pathway in other types of cancer. The KIAA1429 promoter has a p65-binding motif, by which p65 transactivates KIAA1429 expression [27]. SPI1 is an oncogenic transcriptional activator. SPI1 (Spi-1 proto-oncogene) binds to the AGGAAGT region of the KIAA1429 promoter and activates its expression [9].

Gene amplification can drive a higher expression of oncogenic genes in cancer. In lung adenocarcinoma, a study reported that KIAA1429 was amplified in 7.3% of patients, which is significantly higher than METTL3, METTL14, and WTAP, suggesting that KIAA1429 may be the major driver of oncogenesis among these components of the methyltransferase complex [23].

LncRNA *LINC00667* acts as a ceRNA of KIAA1429 mediated by miR-556-5p. KIAA1429 can promote *LINC00667* expression in an m^6^A-dependent manner, which forms a positive feedback loop in breast cancer [49].

USP29 (ubiquitin-specific peptidases 29) deubiquitinates the KIAA1429 protein to prevent ubiquitin-mediated proteasome degradation in colorectal cancer [35].

## 7. Potential Drugs against KIAA1429

A small molecule inhibitor may be used to suppress KIAA1429 function. Rucaparib is a poly ADP-ribose polymerase (PARP) inhibitor. A study showed that rucaparib had a strong interaction with the KIAA1429 protein at Lys1029, Asn1088, and Ala1087 by molecular modeling simulation [25]. Rucaparib treatment could decrease KIAA1429 expression in both mRNA and protein levels, and suppress the proliferation of chronic myeloid leukemia cells. Notably, rucaparib could significantly reduce the resistance of chronic myeloid leukemia to imatinib. Therefore, rucaparib is a promising anti-KIAA1429 drug for cancer therapy [11]. KIAA1429, WTAP, zinc finger CCCH domain-containing protein 13 (ZC3H13), E3 ubiquitin ligase CBLL1 (HAKAI), and RNA binding motif protein 15/15 paralog (RBM15/RBM15B) form a regulatory subunit called the m^6^A -METTL-associated complex (MACOM) that directly binds to RNA substrates and is essential for m^6^A writer activity. A cryo-electron microscopy analysis showed that KIAA1429 and WTAP formed the core structure of the MACOM. KIAA1429 forms a horse-shaped structure with 20 armadillo-like (ARML) repeats and interacts with WTAP extensively [50]. Therefore, interfering with the interaction between KIAA1429 and WTAP may be a potential anti-KIAA1429 method. 

## 8. Conclusions and Remarks

Since the silence of KIAA1429 or the small anti-KIAA1429 molecule inhibitor significantly suppressed tumorigenesis, targeting KIAA1429 may be a potential anti-cancer therapeutic method. KIAA1429 is a central protein for assembling the functional m^6^A methyltransferase complex. Presently, KIAA1429 has not been shown to participate in methylation at other RNA sites than the adenosine N6 position. It is worth developing new methods of disrupting the association between KIAA1429 and other m^6^A writers, which may lead to a reduction in the m^6^A modification of tumor-associated genes.

KIAA1429 is overexpressed in many cancers. However, the expression of KIAA1429 in the tumor microenvironment remains largely unknown. To potentially improve cancer immunotherapy, it is essential to explore the effects of KIAA1429 on cancer immune response beyond its roles in cell proliferation, migration, cell death, and drug resistance. Moreover, the distinct function of KIAA1429 isoforms and the regulatory mechanism of the selection of its polyadenylation sites also need further investigation.

In conclusion, KIAA1429 promotes tumorigenesis and is a promising target for cancer therapy.

## Figures and Tables

**Table 1:** The newly discovered targets of KIAA1429 over the last five years.

Gene Name	Cancer Type	Regulatory Mechanism	Reference
*ARHGAP30*	Lung cancer	Destabilizing *ARHGAP30* mRNAs.	[32]
*BTG2*	Lung cancer	Destabilizing *BTG2* mRNAs.	[23]
*CHST11*	Lung cancer	m^6^A modification on the 3′ UTR of *CHST11* mRNA promotes its degradation.	[10]
*DAPK3*	Lung cancer	m^6^A modification on the 3′ UTR of *DAPK3* mRNA promotes its degradation.	[13]
*ENO1*	Ovarian cancer	Stabilizing *ENO1* mRNAs.	[9]
*FOXM1*	Gastric cancer	m^6^A modificationn on the 3′ UTR of *FOXM1* mRNA increases its stability.	[27]
*GATA3*	Liver cancer	m^6^A modification on the 3′ UTR of *GATA3* mRNAs promotes its degradation.	[19]
*JUN*	Gastric cancer	Stabilizing *JUN* mRNAs.	[20]
*MAP3K2*	Lung cancer	N/A	[29]
*HAS2*	Breast cancer	m^6^A modification on the *HAS1* mRNA increases its stability.	[18]
*HK1*	Liver cancer	m^6^A modification on the *HK1* mRNA increases its stability.	[7]
*HK2*	Colorectal cancer	Stabilizing *HK2* mRNAs.	[33]
*POU6F2-AS1*	Colorectal cancer	m^6^A modification on the *POU6F2-AS1* mRNA increases its stability.	[34]
*RAB27B*	Chronic myeloid leukemia	m^6^A modification on the coding and 3′ UTR of *RAB27B* mRNA increases its stability.	[25]
*RND3*	Liver cancer	m^6^A modification on the 3′ UTR of *RND3* mRNA promotes its degradation.	[6]
*RXFP1*	Lung cancer	Destabilizing *RXFP1* mRNAs.	[24]
*SIRT1*	Colorectal cancer	m^6^A modification on the *SIRT1* mRNA increases its stability.	[11]
*SLC7A11*	Liver cancer	Stabilizing *SLC7A11* mRNAs.	[26]
*SMC1A*	Breast cancer	m^6^A modification on the *SMC1A* mRNA promotes its stability.	[5]
*SOX8*	Colorectal cancer	m^6^A modification on the *SOX8* mRNA promotes its stability.	[35]
*STAT3*	Ewing sarcoma	N/A	[22]
*WTAP*	Lung cancer	m^6^A modification on the 3′ UTR of *WTAP* mRNA increases its stability.	[30]

## Data Availability

All relevant data are within the paper.
